# Ultrasound Doppler renal pulsatility index is a predictive marker of arterial stiffness in children with solitary functioning kidney

**DOI:** 10.1590/2175-8239-JBN-2024-0069en

**Published:** 2025-03-10

**Authors:** Seçil Conkar Tunçay, Gonca Koç, Gülden Hakverdi

**Affiliations:** 1 Ege University, Faculty of Medicine, Department of Pediatric Radiology, Izmir, Turkey.; 2 Cumhuriyet University, Faculty of Medicine, Department of Biostatistics, Sivas, Turkey.

**Keywords:** Solitary Kidney, Vascular Stiffness, Renal Pulsatility Index, Child

## Abstract

**Introduction:**

Patients with solitary functioning kidney (SFK) have glomerular hyperfiltration, hypertension, proteinuria and impaired renal function resulting in microvascular atherosclerotic abnormalities. This condition leads to an increase in arterial stiffness. In this study, we aimed to investigate the usefulness of non-invasive renal Doppler ultrasonography hemodynamic parameters in demonstrating arterial stiffness in pediatric patients with SFK.

**Methods:**

The study included 59 children aged 6–18 years who were diagnosed with SFK. Demographic, biochemical, anthropometric, and blood pressure data were recorded. The renal Doppler ultrasound hemodynamic parameters renal resistive index (RRI), renal pulsatility index (RPI), carotid-femoral pulse wave velocity (cfPWV), central augmentation index (cAIx) and carotid intima media thickness (cIMT) were evaluated.

**Results:**

Eighteen (30.5%) cases with acquired SFK and 41 (64.5%) cases with congenital SFK were detected. Central augmentation indices were higher in children with congenital SFK than in children with acquired SFK (p = 0.038). CkiD-eGFR-SCr-CysC was lower in patients with acquired SFK (p = 0.011). LDL cholesterol levels were higher in children with acquired SFK (p = 0.018). We found a significant correlation between RPI and cfPWV with a correlation coefficient (r) of 0.321 and a statistically significant p-value of 0.013.

**Conclusions:**

Congenital SFK is associated with increased microvascular atherosclerotic burden. RPI assessment with renal Doppler ultrasound may be a non-invasive method to identify arterial stiffness.

## Introduction

Solitary functioning kidney (SFK) is a medical condition in which an individual has only one functional kidney^1^. This condition can be congenital, i.e. the individual is born with an SFK, or acquired, when one of the two kidneys is lost due to surgical removal, injury, disease, or organ donation^2^. In response to a decreased number of nephrons, compensatory mechanisms within the remaining nephrons lead to an augmentation in glomerular perfusion, resulting in glomerular hyperfiltration and the preservation of a consistent estimated glomerular filtration rate (eGFR)^3^. While advantageous in the short term, an elevation in glomerular perfusion (especially glomerular hypertension) may induce deleterious structural alterations in kidney morphology over extended periods. Glomerular hypertension gives rise to glomerulosclerosis in a damaging cycle that reduces the number of functional nephrons.

The well-established association between a reduction in the number of nephrons and various kidney-related conditions, such as SFK, focal segmental glomerulosclerosis, and diabetic nephropathy, encompasses manifestations like hyperfiltration, glomerular hypertension, decline in eGFR, and proteinuria. This linkage underlines the intricate interplay between nephron count and the development of renal pathologies, influencing key parameters indicative of kidney health. According to the hyperfiltration theory, glomerular hypertension acts as an intermediate stage between a low nephron count and a gradual deterioration of renal function. Consequently, indicators of glomerular hypertension, like albuminuria/proteinuria or systemic hypertension, are thought to precede a decline in kidney function. Furthermore, if glomerular hypertension can be averted, this would also stave off the gradual progression of kidney damage^4^, providing a window of opportunity for intervention if detected at an early stage.

Indeed, the vascular endothelium plays a pivotal role in orchestrating various biological processes crucial for vascular health. Its multifaceted functions include the regulation of vascular tone, inflammatory response, thrombosis, and angiogenesis. The endothelium serves as a dynamic interface between the blood and the vessel wall, contributing significantly to the overall homeostasis of the cardiovascular system. Dysregulation of the endothelial function can lead to various vascular disorders and contribute to the pathogenesis of conditions such as atherosclerosis and hypertension. Understanding and monitoring endothelial function is essential in assessing vascular health and identifying potential risk factors for cardiovascular diseases.

Reduced availability of nitric oxide (NO) is a common issue in chronic kidney disease (CKD), particularly end-stage kidney disease (ESKD). This reduction in NO levels in CKD is attributed to various factors that affect the expression and activity of endothelial NO synthase^5^. In clinical studies, researchers have devised both direct and indirect methods to evaluate endothelial dysfunction. Pulse wave velocity (PWV) and amplification index (AIx) are noninvasive techniques used for the evaluation of arterial rigidity, offering crucial insights into vascular well-being and endothelial functionality^6,7^. Additionally, renal resistive index (RRI) and renal artery pulsatility index (RPI) provide insights into the microvascular condition of the renal and intrarenal arteries^8^.

The hypothesis presented in the study suggests that complications associated with SFK, including glomerular hyperfiltration, decreased eGFR, and hypertension, may have an impact on endothelial dysfunction. This hypothesis is based on the premise that the physiological alterations of kidney function observed in SFK, such as increased glomerular perfusion and glomerular hypertension, may contribute to adverse effects on the vascular endothelium.

This study aims to explore the potential correlation between markers of microvascular damage and arterial rigidity, serving as an indicator of atherosclerotic load in individuals with congenital or acquired SFK. By investigating these relationships, the researchers seek to better understand the vascular and renal dynamics in SFK patients, particularly in children aged 6–18 years.

If the hypothesis is supported by the study findings, it could have implications for the early detection and management of endothelial dysfunction in individuals with SFK, potentially offering insights into preventive strategies for cardiovascular and renal complications associated with this medical condition. Therefore, our objective was to assess renal damage indicators including hypertension, decrease in eGFR, hyperfiltration, and proteinuria, as well as parameters of vascular endothelial dysfunction in pediatric patients with congenital or acquired SFK. The findings were then compared between the two groups. Additionally, this study aimed to explore the relationship between renal Doppler ultrasound and markers of microvascular hemodynamics, i.e. RRI and RPI, which serve as indicators of atherosclerotic burden. Based on our results, we anticipate that noninvasive examinations of microvascular status will facilitate early detection of renal damage in children with SFK, which in turn may slow down the development of CKD and ESRD thanks to preventive treatments.

## Methods

The study included fifty-nine children aged 6 to 18 years with either acquired or congenital SFK. These children were under follow-up at the Ege University Children’s Hospital Pediatric Nephrology Clinic. Among the participants, 30.5% (18 patients) had acquired SFK, which resulted from nephrectomy, while 64.5% (41 patients) had congenital SFK. The inclusion of both acquired and congenital cases allowed for a comprehensive examination of the vascular and renal characteristics in children with SFK considering the different etiologies.

The study was conducted from March 2021 to June 2022. The research protocol received approval from the local ethics committee (decision number: 99166796-050.06.04). To ensure ethical standards, written informed consent was obtained from the guardians of all participants. Additionally, eligible participants were provided with information about the study’s objectives, allowing them to make informed decisions regarding their involvement. The adherence to ethical guidelines ensures the protection of participants’ rights and welfare throughout the research process. SFK was characterized by a unilateral absence of a viable kidney determined by ultrasound and renal scintigraphy. Exclusion criteria included individuals with deficient renal uptake or confirmed renal scarring, as identified in the Tc99 m-dimercaptosuccinic acid scan (Tc99 m-DMSA). Other exclusion criteria involved conditions such as vesicoureteral reflux, ureteropelvic junction obstruction, duplex system, megaureter, posterior urethral valve, or neurogenic bladder. Additionally, individuals diagnosed with type 1 or type 2 diabetes mellitus and children with an eGFR less than 30 mL/min/1.73m^2^ were not included in the study. These criteria were established to ensure the homogeneity of the study population and focus on patients with SFK without confounding factors from other underlying conditions. Renal agenesis and multicystic dysplastic kidney (MCDK) were accepted as congenital SFK. Acquired SFK was defined as the state of having only one functional kidney due to dysfunction of the contralateral kidney resulting from various kidney diseases or nephrectomy^9^.

Anthropometric data were collected from all children. Blood samples were collected from the participants for various biochemical analyses. The measurements included serum creatinine (SCr), relevant enzymes, blood urea nitrogen (BUN), C-reactive protein (CRP), erythrocyte sedimentation rate (ESR), high-density lipoprotein (HDL), low-density lipoprotein (LDL), total cholesterol, triglyceride levels, and serum cystatin C (CysC).

Cystatin C levels were assessed using the immunonephelometric method with the Siemens-BNTM II nephelometer device. The immunonephelometric method is a technique used for quantitative determination of specific proteins using antibodies to detect and quantify the target protein, providing precise and reliable results. First morning urine samples were obtained from 59 children. Urine protein/creatinine ratio >0.2 mg/mg was defined as proteinuria. We calculated eGFR by the CkiD-eGFR-SCr-CysC formula^10,11^. An eGFR>120 mL/min/1.73m^2^ was defined as hyperfiltration^12^. Systolic (SBP) and diastolic (DBP) blood pressure was measured with an oscillometric method using an OMRON automatic blood pressure monitor. Blood pressure (BP) was measured after 5 minutes of rest. Hypertension was defined as systolic BP and/or diastolic BP z-score greater than 1.65 SDS (95th percentile) adjusted for sex, age, and height^13^. cfPWV was measured with a Vicorder (Skidmore Medical Limited, Bristol, UK) device. For the evaluation of arterial stiffness parameters, cfPWV, and cAIx, patients fasted for 12 hours and rested comfortably in the supine position for 30 minutes.

Peripheral and central arterial pulse waveforms were recorded from radial and carotid arteries by means of a Vicorder device. Mean values of the compound radial waveforms were calculated using the computer program developed specifically for this study.

The cfPWV was estimated as follows: cfPWV (m/s) = 0.049 × age (years) + 0.008 × height (cm) + 0.024 × mean arterial pressure (MAP) (mm Hg) + 1.129^14^.

All cIMT measurements were performed by a single pediatric radiologist (G.K.) with ten years of post-residency experience in vascular ultrasound. Accuson S2000^®^ ultrasound system, Siemens, and 14L5 linear array probe were used for the cIMT measurements. In the study, certain measurements such as cIMT and cfPWV were repeated three times for each participant, and the average value of these measurements were used for further analysis.

To assess cIMT and cfPWV, z-scores were calculated. Z-scores are statistical measures that express the standard deviation of a raw score from the group mean. In this case, z-scores were based on specific median (M), skewness (L), and coefficient of variation (S) values adjusted for the child’s height.

Values above 1.65 standard deviations (SDS), corresponding to the 95th percentile, according to sex and height in healthy children, were used to define increased cIMT and cfPWV. This approach helps identify values that fall outside the normal range and may indicate abnormal thickness in the carotid intima-media or increased pulse wave velocity, both of which can be indicative of vascular issues^15,16^.

RRI and RPI were assessed with the patients positioned on their sides using spectral renal Doppler US probes placed on the surface over the arcuate arteries coursing along the corticomedullary junction. In the longitudinal plane, three consecutive measurements were performed on the upper and lower poles and in the mid zone of the SFK. Mean RRI and RPI values were then obtained.

### Statistical Analyzes

The statistical analyses were conducted using the IBM SPSS Statistics 25.0 program. The normality of continuous variables was assessed using the Shapiro-Wilk test. For variables that exhibited a normal distribution, the results were presented as mean ± standard deviation (mean ± SD). In contrast, variables with a non-normal distribution were expressed as median (minimum-maximum) values. This approach provides a comprehensive representation of the central tendency and variability of the data, considering the distribution characteristics of each variable. Categorical data were presented as numbers and percentages (%).

To compare paired groups, the Student t-test or the Mann-Whitney U test were used depending on the nature of the data distribution. Chi-square or Pearson test was deployed as appropriate for the comparison of categorical variables. The correlation between variables was evaluated by Spearman correlation analysis. The threshold for statistical significance was established as p < 0.05 for all analyzes.

## Results

A total of 59 cases were included, comprising 31 (52.5%) male and 28 (47.5%) female patients. The mean age of the participants was 10.78 ± 4.47 years, with an age range spanning from 6 to 18 years. Eighteen (30.5%) cases were diagnosed as MCDK, 23 (38.9%) as congenital SFK, and 18 (30.5%) as SFK caused by other etiologic factors. Eighteen (30.5%) cases with acquired SKF and 41 (64.5%) cases with congenital SFK were detected. The two groups (congenital and acquired SFK) did not show statistically significant differences in age, serum BUN, triglyceride, total cholesterol, high-density lipoprotein, CRP levels, weight SDS, height SDS, body mass index (BMI), blood pressure, urine protein/creatinine ratio, and erythrocyte sedimentation rate. However, there was a statistically significant difference in serum creatinine levels, with higher levels observed in patients with acquired SFK (p = 0.023). This suggests a potential distinction in renal function between the congenital and acquired SFK groups (Table 1).

**Table 1:** Demographic and laboratory data of patients with solitary functioning kidney

Parameters	Congenital SFK (n = 41)	Acquired SFK (n = 18)	p^a,b,c^
Age (years)	10.12 ± 4.45	12.28 ± 4.28	0.089^a^
Gender			
Male, n (%)	24 (58.5%)	7 (38.9%)	0.164^b^
Female, n (%)	17 (41.5%)	11 (61.1%)	
Height z-score	−0.18 ± 2.48	−0.56 ± 2.09	0.57^a^
Weight z-score	−0.03 ± 2.05	0.16 ± 1.44	0.727^a^
Body mass index (kg/m^2^)	18.89 ± 4.85	20.95 ± 7.48	0.210^a^
Body mass index z-score	0.37 ± 1.39	0.21 ± 1.51	0.689^a^
Systolic BP	112.98 ± 13.14	116.11 ± 9.32	0.364^a^
Diastolic BP	71.10 ± 9.75	73.39 ± 9.25	0.402^a^
Systolic BP z-score	1.25 ± 1.20	1.23 ± 0.81	0.959^a^
Diastolic BP z-score	1.25 ± 0.87	1.13 ± 0.73	0.618^a^
Blood urea nitrogen (mg/dL)	26.63 ± 10.74	28.87 ± 21.83	0.599^a^
Serum creatinine (mg/dL)	0.53 (0.27–1.20)	0.70 (0.49–1.62)	**0.010^c^**
eGFR (ml/min/1.73m^2^)	103.07 ± 26.00	84.78 ± 20.52	**0.011^a^**
Triglyceride (mg/dL)	91.78 ± 63.87	94.28 ± 52.22	0.885^a^
Total cholesterol (mg/dL)	146.90 ± 28.39	161.56 ± 30.77	0.080^a^
High-density lipoprotein (mg/dl)	57.24 ± 18.42	55.94 ± 15.68	0.795^a^
LDL cholesterol (mg/dL)	75.0 (16–140)	92.50 (47–262)	**0.005^c^**
C-reactive protein (mg/dL)	1.74±3.96	0.82±0.80	0.331^a^
Erythrocyte sedimentation rate (mm/h)	6.29 ± 5.49	6.11 ± 5.71	0.908^a^
Urine protein/creatinine ratio (mg/mg)	0.16 ± 0.18	0.22 ± 0.36	0.558^a^

Abbreviations – SFK: Solitary functioning kidney; BP: blood pressure; LDL; low-density lipoprotein; eGFR: estimated glomerular filtration rate. Notes – Numerical data are presented as mean ± SD and median (minimum-maximum) while categorical data as numbers and percentages. P value is used for the comparisons between congenital and acquired solitary functioning kidney patients and p < 0.05 indicates statistical significance.

^a^Student t test.

^b^Chi-square or Pearson test when appropriate.

^c^Mann-Whitney U test.

**Figure 1.**
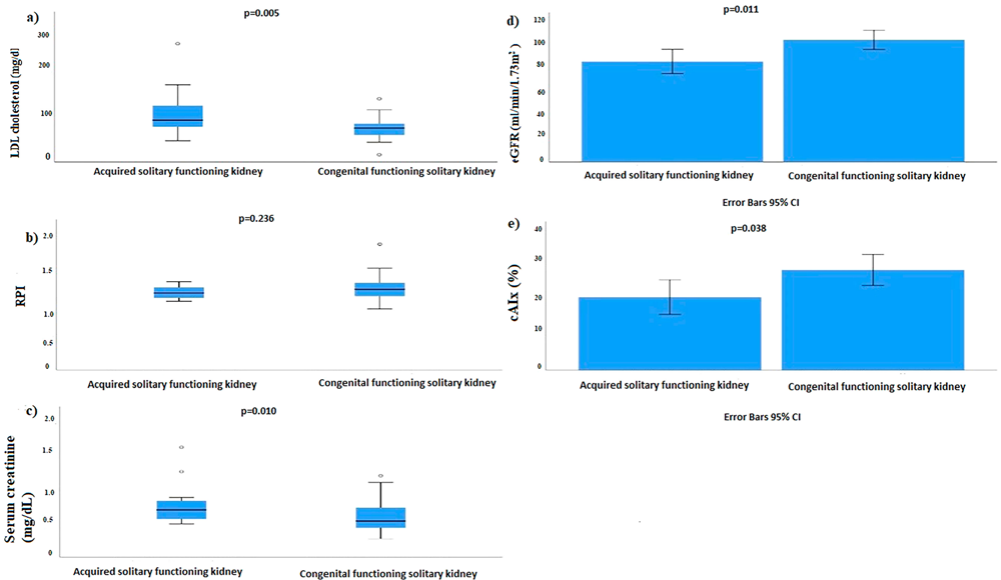
(A) Low-density lipoprotein (LDL) cholesterol (mg/dL), (B) Pulsatility Index, (C) Serum creatinine (mg/dL), (D) estimated glomerular filtration rate (eGFR) (mL/min/1.73 m^2^), (E) Central augmentation indices (%) in congenital functioning solitary kidney and acquired solitary functioning kidney patients.

In addition, CkiD-eGFR-SCr-CysC was lower (p = 0.011) and LDL cholesterol levels were higher (p = 0.018) in children with acquired SFK (Figure 1).

The number of cases with hypertension, proteinuria, eGFR decline, and hyperfiltration, as indicators of renal injury, were not significantly difference between groups (p > 0.005 for all). However, hyperfiltration was significantly more common in congenital SFK patients (p = 0.038) (Table 2).

**Table 2:** Renal injury parameters associated with solitary functioning kidney

Clinical parameters	Congenital SFK (n = 41)	Acquired SFK (n = 18)	p^a^
Hypertension, (n, %)	19 (46.3)	4 (22.2)	0.080
Proteinuria, (n, %)	4 (9.8)	4 (22.2)	0.198
eGFR < 90 mL/min/1.73m^2^, (n, %)	17 (41.5)	13 (72.2)	0.061
Hyperfiltration (eGFR > 2SDS) (n, %)	21 (51.2)	4 (22.2)	**0.038**

Abbreviations – SFK: Solitary functioning kidney; eGFR: estimated glomerular filtration rate. Notes – Data are presented as numbers and percentages. P value is used for the comparisons between congenital and acquired solitary functioning kidney patients and p < 0.05 indicates statistical significance.

^a^Chi-square test or Fisher’s exact test was used as appropriate.

There was no statistically significant difference between groups in terms of cIMT, cfPWV, PWV, height-z, and cIMT height-z values. cAIx was found to be significantly higher in the congenital SFK than in the acquired SFK (p = 0.038) group (Table 3).

**Table 3:** Vascular endothelial dysfunction parameters expressed in children with congenital and acquired solitary functioning kidney

Doppler parameters	Congenital SFK (n = 41)	Acquired SFK (n = 18)	p^a,b^
cIMT	0.48 ± 0.08	0.51 ± 0.10	0.402^a^
cfPWV	4.55 ± 0.41	4.56 ± 0.36	0.952^a^
cAIx (%)	28.34 ± 13.88	20.67 ± 9.86	**0.038^a^**
cfPWV height z-score	0.19 ± 0.47	0.27 ± 0.35	0.511^a^
cIMT height z-score	2.60 ± 1.92	2.89 ± 2.29	0.624^a^
RRI	0.63 ± 0.04	0.63 ± 0.03	0.777^a^
RPI	1.19 (0.9–1.86)	1.14 (1.01–1.30)	0.236^b^

Abbreviations – cIMT; Carotid intima-media thickness; cfPWV: carotid-femoral pulse wave velocity; cAIx: central augmentation index; RRI: renal resistive index; RPI: renal artery pulsatility index. Notes – Numerical data are presented as mean ± SD and median (minimum-maximum). p value is used for the comparisons between congenital and acquired solitary functioning kidney patients and p < 0.05 indicates statistical significance.

^a^Student t-test.

^b^Mann-Whitney U-test.

No correlation was found between RRI and cAIx, cfPWV *z* height, cIMT *z* height, cfPWV (p = 0.236, p = 0.94, p = 0.47, and p = 0.109, respectively). There was a positive correlation between RPI and cfPWV (r = 0.321 and p = 0.013) (Table 4).

**Table 4:** Relationship between renal resistive index, renal artery pulsatility index and vascular endothelial dysfunction parameters

Parameters	RRI			RPI	
	r	p^a^		r	p^a^
cAIx (%)	0.157	0.236		0.51	0.71
cfPWV z height	0.009	0.94		0.148	0.26
cIMT z height	0.96	0.47		0.071	0.59
cfPWV	0.211	0.109		0.321	**0.013**

Abbreviations – RRI: Renal resistive index; RAPI: renal artery pulsatility index; cAIx: central augmentation index; cfPWV: carotid-femoral pulse wave velocity; cIMT: carotid intima-media thickness. Note – p < 0.05 indicates statistical significance.

^a^Spearman’s correlation analysis.

## Discussion

The study has identified arterial stiffness and indications of renal damage associated with renal endothelial dysfunction in children diagnosed with congenital or acquired SFK. This suggests a potential link between microvascular damage and arterial rigidity in individuals with SFK, highlighting the importance of assessing these factors in pediatric patients with SFK. The study contributes to understanding the vascular and renal implications of SFK in the pediatric population.

In our study, we have found that children with acquired SFK had higher serum creatinine and lower eGFR values. The study findings indicate that the decline in eGFR was more significant in the acquired SFK group compared to the congenital SFK group. This observation aligns with the research by Abou Jaoude et al.^17^, who also reported an inverse relationship between GFR and follow-up time in patients with acquired SFK, while not observing the same pattern in cases of congenital SFK. The differences in the patterns of decline may suggest distinct mechanisms or responses to SFK in congenital versus acquired cases. In our study, eGFR was calculated according to the CkiD-eGFR-SCr-CysC formula, the use of which is recommended in studiess^11^ Individuals with congenital SFK undergo a process of generating extra nephrons at an early stage, which alleviates kidney damage^18,19^. On the contrary, research has demonstrated that in cases of acquired SFK, a compensatory response typically manifests a few years after nephrectomy^20^. In our study, consistent with the reports from previous studies, we have found that eGFR was lower in children with acquired SFK. In addition, LDL cholesterol was found to be higher in cases with acquired SFK. Based on these findings, it can be concluded that LDL levels tend to increase with age, considering that the group with acquired SFK was older on average.

Patients with congenital and acquired SFK are at risk of hypertension, hyperfiltration, azotemia and proteinuria due to decreased number of nephrons^21^. The study notes that the reported prevalence of hypertension in SFK patients is 65%, as determined by normal office blood pressure and ambulatory blood pressure monitoring. However, in the current study, there was no significant difference between the two groups in terms of the presence of proteinuria and hypertension. Interestingly, the frequency of hypertension in congenital SFK patients was higher (46%) compared to the literature. This variation may indicate potential differences in the prevalence and characteristics of hypertension among SFK patients, particularly those with a congenital origin, compared to existing literature findings. Individuals with congenital or acquired SFK are susceptible to hyperfiltration and a decline in eGFR. This state of hyperfiltration develops as a result of a compensatory renal hypertrophy. In congenital SFK, the process of compensatory renal hypertrophy is typically completed during the intrauterine period in around 90% of cases. In acquired SFK, compensatory hypertrophy occurs after nephrectomy^20^, but it may not be sufficient.

The compensatory hypertrophy process induces glomerular cell activation, fibrosis, vasoconstriction, and ultimately a decline in eGFR. The findings are in accordance with the KIdney of MONofunctional Origin (KIMONO) study, which also observed a higher incidence of renal injury in the acquired SFK group compared to the congenital SFK group. Renal injury in this context was defined as hypertension, proteinuria, an impaired eGFR, or the use of renoprotective medication. This finding aligns with our observations, where children with acquired SFK exhibited higher serum creatinine levels and a more pronounced decline in eGFR compared to those with congenital SFK. These outcomes suggest that the process of acquired SFK may contribute to differences in renal outcomes and complications^18^. Additionally, the researchers observed that the acquired SFK group had a higher proportion of impaired eGFR cases compared to the congenital SFK group^18,22^. Consistent with previous studies, in our study, eGFR was observed to be lower in the acquired SFK cases, which we believed that it was related to the delayed activation of nephron adaptation mechanisms. Similarly, another study showed that children with SFK have particularly high morbidity^23^.

The development of hyperfiltration in children with congenital SFK may be the result of arterial stiffness. Besides, smoking, aging, hypercholesterolemia, hypertension, hyperglycemia and family history of early atherosclerotic disease, obesity, high CRP, and chronic systemic infection are also associated with arterial stiffness^24^. Arterial stiffness, a known risk factor for cardiovascular issues, can be assessed noninvasively using methods like pulse wave analysis. The cAIx is a measure of pulse wave reflection and is a predictor of cardiovascular events^25^. In our study, the cAIx was found to be significantly higher in the congenital SFK group than in the acquired SFK group.

The study confirms a relevant finding from another study that found an associating between an increased cfPWV and declining kidney function in both diabetic and non-diabetic populations^25^. This underscores the importance of assessing arterial stiffness, particularly through measures like cfPWV, as an indicator of cardiovascular health and its potential connection to kidney function. Arterial stiffness, as measured by cfPWV, is considered a risk factor for cardiovascular events, and its correlation with declining kidney function suggests an intricate relationship between cardiovascular health and renal outcomes^26^. SFK tries to compensate for the increased glomerular blood flow and the loss of function by increasing the workload leading to hyperfiltration, which increases the risk of hypertension, microalbuminuria, and impaired renal functions^27^. The cfPWV is an independent predictor of cardiovascular and all-cause mortality^28^. The study recommends cfPWV as the gold standard for assessing arterial stiffness due to its clinical applicability, strong physiological basis, and relevance to clinical outcomes. cfPWV is as a reliable method to evaluate arterial stiffness, providing valuable insights into cardiovascular health^29^.

The cIMT is a morphological parameter reflecting structural changes in the arterial wall that may lead to arteriosclerosis. The study found that cAIx was significantly higher in children with congenital SFK. Additionally, a significant negative correlation was detected between RPI and cfPWV. These findings suggest that there may be subclinical unfavorable changes in arterial function, particularly in children with congenital SFK. The cAIx index, which reflects pulsewave reflection and is a predictor of cardiovascular events, was elevated in congenital SFK, indicating potential arterial stiffness. The positive correlation between RPI and cfPWV suggests an association between renal microvascular status and arterial stiffness.

It has been suggested that RRI and RPI measured by Doppler ultrasound reflect intrarenal vascular resistance and that the increase in RRI of the renal arteries is associated with the severity of systemic atherosclerosis^30,31^. RRI increases in renal parenchymal diseases. Our study included patients without renal parenchymal disease^32^. RPI increases when arterial stiffness increases. The vascular resistance index (RRI) and pulsatility index (RPI) measure the elasticity of arterial vascular branches. Therefore, the results of our study showed that RPI increased as cfPWV (arterial stiffness) increased. The fact that the cAIx index is found to be significantly higher in children with congenital SFK may indicate the onset of arterial stiffness in these children. This implies a potential relationship between arterial stiffness and microvascular resistance in the renal arteries. In the light of this information, RPI, which is an easier, more accessible, and noninvasive evaluation criterion in pediatric patients with SFK, may become a new arterial stiffness evaluation method. The inclusion of cfPWV as a marker of arterial stiffness, as well as hypertension, GFR, and proteinuria, may be considered in the follow-up of pediatric patients with SFK.

Although the number of patients in our study was small, this is the first study in the literature showing that renal microvascular status can be an indicator of arterial stiffness. However, we believe that randomized controlled prospective studies involving larger groups of patients should be performed to confirm these findings.

Our study had some limitations including the small number of patients with acquired SFK. A larger sample would provide reliable estimates of deteriorated renal functions. Besides, microalbuminuria, which is a more sensitive indicator of kidney damage than proteinuria, should be evaluated, and the duration of acquired SFK should be indicated.

In conclusion, we found that subclinical unfavorable changes in arterial function in children with SFK begin in the very early stages of childhood, but kidney damage develops later in childhood and RPI is associated with vascular endothelial dysfunction.
