# Supplementary material for: Ultrasound Doppler renal pulsatility index is a predictive marker of arterial stiffness in children with solitary functioning kidney
Source: J Bras Nefrol. 2025 Mar 10;47(2):e20240069. doi: 10.1590/2175-8239-JBN-2024-0069en (PMC11924956; doi:10.1590/2175-8239-JBN-2024-0069en)
Supplement: Translation

# Translation: Índice de pulsatilidade renal por ultrassonografia Doppler como um marcador preditivo de rigidez arterial em crianças com rim solitário funcional

**DOI:** 10.1590/2175-8239-JBN-2024-0069pt

**Authors:** Seçil Conkar Tunçay, Gonca Koç, Gülden Hakverdi

**Affiliations:** 1 Ege University, Faculty of Medicine, Department of Pediatric Radiology, Izmir, Turquia.; 2 Cumhuriyet University, Faculty of Medicine, Department of Biostatistics, Sivas, Turquia.

**Keywords:** Rim Único, Rigidez Vascular, Índice de Pulsatilidade Renal, Criança

## Abstract

**Introdução:**

Pacientes com rim solitário funcional (RSF) apresentam hiperfiltração glomerular, hipertensão, proteinúria e função renal prejudicada, resultando em anormalidades ateroscleróticas microvasculares. Essa condição leva a um aumento na rigidez arterial. Neste estudo, nosso objetivo foi investigar a utilidade dos parâmetros hemodinâmicos obtidos por ultrassonografia renal com Doppler não invasiva na demonstração da rigidez arterial em pacientes pediátricos com RSF.

**Métodos:**

O estudo incluiu 59 crianças com idades entre 6 e 18 anos, diagnosticadas com RSF. Foram registrados dados demográficos, bioquímicos, antropométricos e de pressão arterial. Foram avaliados os parâmetros hemodinâmicos da ultrassonografia renal com Doppler, como o índice de resistividade renal (IRR), índice de pulsatilidade renal (IPR), velocidade da onda de pulso carotídeo-femoral (VOPcf), índice de amplificação central (cAIx) e espessura da íntima média carotídea (EIMc).

**Resultados:**

Foram detectados 18 (30,5%) casos com RSF adquirido e 41 (64,5%) casos com RSF congênito. Os índices de amplificação central foram maiores em crianças com RSF congênito do que naquelas com RSF adquirido (p = 0,038). CkiD-eGFR-SCr-CysC foi menor em pacientes com RSF adquirido (p = 0,011). Os níveis de colesterol LDL foram maiores em crianças com RSF adquirido (p = 0,018). Encontramos uma correlação significativa entre o IPR e a VOPcf com um coeficiente de correlação (r) de 0,321 e um valor de p estatisticamente significativo de 0,013.

**Conclusões:**

O RSF congênito está associado ao aumento da carga aterosclerótica microvascular. A avaliação do IPR por ultrassonografia Doppler renal pode ser um método não invasivo para identificar a rigidez arterial.

## Introdução

O rim solitário funcional (RSF) é uma condição médica na qual o indivíduo possui apenas um rim funcional^1^. Essa condição pode ser congênita, ou seja, o indivíduo nasce com um RSF, ou adquirida, quando um dos dois rins é perdido devido à remoção cirúrgica, lesão, doença ou doação de órgão^2^. Em resposta a um número reduzido de néfrons, mecanismos compensatórios dentro dos néfrons remanescentes levam a um aumento na perfusão glomerular, resultando em hiperfiltração glomerular e preservação de uma taxa de filtração glomerular estimada (TFGe) consistente^3^. Embora vantajoso em curto prazo, uma elevação na perfusão glomerular (especialmente a hipertensão glomerular) pode induzir alterações estruturais deletérias na morfologia renal durante períodos prolongados. A hipertensão glomerular dá origem à glomeruloesclerose em um ciclo prejudicial que reduz o número de néfrons funcionais.

A associação bem estabelecida entre uma redução no número de néfrons e diversas condições relacionadas aos rins, como o RSF, a glomeruloesclerose segmentar focal e a nefropatia diabética, engloba manifestações como hiperfiltração, hipertensão glomerular, declínio na TFGe e proteinúria. Essa ligação ressalta a intrincada interação entre a contagem de néfrons e o desenvolvimento de patologias renais, influenciando parâmetros-chave indicativos da saúde renal. De acordo com a teoria da hiperfiltração, a hipertensão glomerular atua como um estágio intermediário entre uma baixa contagem de néfrons e a deterioração gradual da função renal. Consequentemente, acredita-se que os indicadores de hipertensão glomerular, como albuminúria/proteinúria ou hipertensão sistêmica, precedem um declínio na função renal. Além disso, se a hipertensão glomerular puder ser evitada, isso também evitaria a progressão gradual do dano renal^4^, proporcionando uma janela de oportunidade para intervenção, caso detectada em um estágio inicial.

De fato, o endotélio vascular desempenha um papel fundamental na orquestração de diversos processos biológicos cruciais para a saúde vascular. Suas funções multifacetadas incluem a regulação do tônus vascular, a resposta inflamatória, a trombose e a angiogênese. O endotélio funciona como uma interface dinâmica entre o sangue e a parede do vaso, contribuindo de maneira significativa para a homeostase geral do sistema cardiovascular. A desregulação da função endotelial pode levar a diversos distúrbios vasculares e contribuir para a patogênese de condições como a aterosclerose e a hipertensão. Compreender e monitorar a função endotelial é essencial na avaliação da saúde vascular e na identificação de potenciais fatores de risco para doenças cardiovasculares.

A disponibilidade reduzida de óxido nítrico (NO) é um problema comum na doença renal crônica (DRC), especialmente na doença renal em estágio terminal (DRET). Essa redução nos níveis de NO na DRC é atribuída a vários fatores que afetam a expressão e a atividade da NO sintase endotelial^5^. Em estudos clínicos, os pesquisadores criaram métodos diretos e indiretos para avaliar a disfunção endotelial. A velocidade da onda de pulso (VOP) e o índice de amplificação (AIx) são técnicas não invasivas utilizadas para avaliar a rigidez arterial, oferecendo percepções cruciais sobre o bem-estar vascular e a funcionalidade endotelial^6,7^. Além disso, o índice de resistividade renal (IRR) e o índice de pulsatilidade da artéria renal (IPR) fornecem informações sobre a condição microvascular das artérias renais e intrarrenais^8^.

A hipótese apresentada no estudo sugere que as complicações associadas ao RSF, incluindo hiperfiltração glomerular, redução da TFGe e hipertensão, podem ter impacto na disfunção endotelial. Essa hipótese baseia-se na premissa de que as alterações fisiológicas da função renal observadas no RSF, como aumento da perfusão glomerular e hipertensão glomerular, podem contribuir para efeitos adversos no endotélio vascular.

O objetivo deste estudo é explorar a possível correlação entre marcadores de dano microvascular e rigidez arterial, servindo como um indicador de carga aterosclerótica em indivíduos com RSF congênito ou adquirido. Ao investigar essas relações, os pesquisadores buscam compreender melhor as dinâmicas vascular e renal em pacientes com RSF, especialmente em crianças de 6 a 18 anos de idade.

Se a hipótese for apoiada pelos achados do estudo, ela pode ter implicações para a detecção precoce e o manejo da disfunção endotelial em indivíduos com RSF, possivelmente oferecendo *insights* sobre estratégias preventivas para complicações cardiovasculares e renais associadas a essa condição médica. Portanto, nosso objetivo foi avaliar indicadores de dano renal, incluindo hipertensão, diminuição da TFGe, hiperfiltração e proteinúria, bem como parâmetros de disfunção endotelial vascular em pacientes pediátricos com RSF congênito ou adquirido. Os achados foram então comparados entre os dois grupos. Além disso, esta análise teve como objetivo explorar a relação entre a ultrassonografia Doppler renal e os marcadores da hemodinâmica microvascular, ou seja, IRR e IPR, que servem como indicadores de carga aterosclerótica. Com base em nossos resultados, nossa expectativa é que os exames não invasivos do estado microvascular facilitem a detecção precoce de danos renais em crianças com RSF, o que, por sua vez, pode retardar o desenvolvimento de DRC e DRET graças a tratamentos preventivos.

## Métodos

O estudo incluiu 59 crianças com idades entre 6 e 18 anos com RSF adquirido ou congênito. Essas crianças estavam sob acompanhamento na Clínica de Nefrologia Pediátrica do *Ege University Children’s Hospital*. Entre os participantes, 30,5% (18 pacientes) apresentaram RSF adquirido, resultante de nefrectomia, enquanto 64,5% (41 pacientes) apresentaram RSF congênito. A inclusão de casos adquiridos e congênitos permitiu uma análise abrangente das características vasculares e renais em crianças com RSF, considerando as diferentes etiologias.

O estudo foi realizado de março de 2021 a junho de 2022. O protocolo de pesquisa recebeu aprovação do comitê de ética local (número da decisão: 99166796-050.06.04). Para garantir os padrões éticos, obteve-se o consentimento informado por escrito dos responsáveis de todos os participantes. Além disso, os participantes elegíveis receberam informações sobre os objetivos do estudo, o que lhes permitiu tomar decisões informadas quanto ao seu envolvimento. A adesão às diretrizes éticas garante a proteção dos direitos e do bem-estar dos participantes ao longo de todo o processo de pesquisa. O RSF foi caracterizado pela ausência unilateral de um rim viável, determinada por ultrassonografia e cintilografia renal. Os critérios de exclusão incluíram indivíduos com captação renal deficiente ou cicatriz renal confirmada, conforme identificado na cintilografia com ácido Tc99 m-dimercaptosuccínico (Tc99 m-DMSA). Outros critérios de exclusão envolveram condições como refluxo vesicoureteral, obstrução da junção ureteropélvica, sistema duplex, megaureter, válvula uretral posterior ou bexiga neurogênica. Além disso, não foram incluídos no estudo indivíduos diagnosticados com *diabetes mellitus* tipo 1 ou tipo 2 e crianças com TFGe inferior a 30 mL/min/1,73m^2^. Esses critérios foram estabelecidos para garantir a homogeneidade da população do estudo e focar em pacientes com RSF sem fatores de confusão decorrentes de outras condições subjacentes. A agenesia renal e o rim displásico multicístico (RDM) foram aceitos como RSF congênito. O RSF adquirido foi definido como o estado de ter apenas um rim funcional devido à disfunção do rim contralateral, resultante de diversas doenças renais ou nefrectomia^9^.

Foram coletados dados antropométricos de todas as crianças. Amostras de sangue foram coletadas dos participantes para diversas análises bioquímicas. As medições incluíram creatinina sérica (CrS), enzimas relevantes, nitrogênio ureico no sangue (BUN), proteína C-reativa (PCR), velocidade de hemossedimentação (VHS), lipoproteína de alta densidade (HDL), lipoproteína de baixa densidade (LDL), colesterol total, níveis de triglicerídeos e cistatina C sérica (CysC).

Os níveis de cistatina C foram avaliados pelo método de imunonefelometria com o dispositivo nefelômetro Siemens-BNTM II. O método de imunonefelometria é uma técnica utilizada para a determinação quantitativa de proteínas específicas usando anticorpos para detectar e quantificar a proteína-alvo, fornecendo resultados precisos e confiáveis. Foram obtidas amostras da primeira urina da manhã de 59 crianças. A relação proteína/creatinina urinária >0,2 mg/mg foi definida como proteinúria. Calculamos a TFGe pela fórmula CkiD-eGFR-SCr-CysC^10,11^. Uma TFGe>120 mL/min/1,73m^2^ foi definida como hiperfiltração^12^. A pressão arterial sistólica (PAS) e diastólica (PAD) foram medidas pelo método oscilométrico utilizando um monitor de pressão arterial automático OMRON. A pressão arterial (PA) foi medida após 5 minutos de repouso. A hipertensão foi definida como um escore z de PA sistólica e/ou diastólica superior a 1,65 DP (percentil 95) ajustado para sexo, idade e altura^13^. A VOPcf foi medida com um dispositivo Vicorder (*Skidmore Medical Limited*, Bristol, Reino Unido). Para a avaliação dos parâmetros de rigidez arterial, VOPcf e cAIx, os pacientes permaneceram em jejum por 12 horas e descansaram confortavelmente em posição supina por 30 minutos.

As formas da onda de pulso arterial periférica e central foram registradas a partir das artérias radial e carótida por meio de um dispositivo Vicorder. Os valores médios das formas de onda radiais compostas foram calculados utilizando o programa de computador desenvolvido especificamente para este estudo.

A VOPcf foi estimada da seguinte forma: VOPcf (m/s) = 0,049 × idade (anos) + 0,008 × altura (cm) + 0,024 × pressão arterial média (PAM) (mm Hg) + 1,129^14^.

Todas as medições de EIMc foram realizadas por um único radiologista pediátrico (G.K.) com dez anos de experiência pós-residência em ultrassonografia vascular. O sistema de ultrassom Accuson S2000^®^ da Siemens e a sonda 14L5 de matriz linear foram utilizados para as medições da EIMc. No estudo, determinadas medições, como EIMc e VOPcf, foram repetidas três vezes para cada participante, e o valor médio dessas medidas foi utilizado para análise posterior.

Para avaliar a EIMc e a VOPcf, foram calculados os escores z. Os escores Z são medidas estatísticas que expressam o desvio padrão de um escore bruto da média do grupo. Nesse caso, os escores z foram baseados em valores específicos de mediana (M), assimetria (L) e coeficiente de variação (S) ajustados para a altura da criança.

Valores superiores a 1,65 desvios-padrão (DP), correspondentes ao percentil 95, de acordo com o sexo e a altura em crianças saudáveis, foram usados para definir o aumento da EMIc e da VOPcf. Essa abordagem ajuda a identificar valores fora da faixa normal e podem indicar espessamento anormal da íntima-média carotídea ou aumento da velocidade da onda de pulso, ambos podendo ser indicativos de problemas vasculares^15,16^.

A avaliação do IRR e do IPR foi feita com os pacientes posicionados de lado, utilizando sondas de US Doppler renal espectral colocadas na superfície sobre as artérias arqueadas que percorrem a junção corticomedular. No plano longitudinal, foram realizadas três medições consecutivas nos polos superior e inferior e na zona média do RSF. Os valores médios de IRR e IPR foram então obtidos.

### Análises Estatísticas

As análises estatísticas foram realizadas com o programa IBM SPSS Statistics 25.0. A normalidade das variáveis contínuas foi avaliada com o teste de Shapiro-Wilk. Para as variáveis que exibiram distribuição normal, os resultados foram apresentados como média ± desvio padrão (média ± DP). Em contrapartida, as variáveis com uma distribuição não normal foram expressas como valores medianos (mínimo-máximo). Essa abordagem fornece uma representação abrangente da tendência central e da variabilidade dos dados, considerando as características de distribuição de cada variável. Os dados categóricos foram apresentados como números e porcentagens (%).

Para comparar grupos pareados, foram utilizados o teste t de Student ou o teste U de Mann-Whitney, dependendo da natureza da distribuição dos dados. O teste qui-quadrado ou de Pearson foi empregado, conforme apropriado, para a comparação de variáveis categóricas. A correlação entre as variáveis foi avaliada pela análise de correlação de Spearman. O limite para significância estatística foi estabelecido como p < 0,05 para todas as análises.

## Resultados

Um total de 59 casos foi incluído, compreendendo 31 (52,5%) pacientes do sexo masculino e 28 (47,5%) do sexo feminino. A média de idade dos participantes foi de 10,78 ± 4,47 anos, com uma faixa etária que variou de 6 a 18 anos. Dezoito (30,5%) casos foram diagnosticados como RDM, 23 (38,9%) como RSF congênito e 18 (30,5%) como RSF causado por outros fatores etiológicos. Foram detectados 18 (30,5%) casos com RSF adquirido e 41 (64,5%) casos com RSF congênito. Os dois grupos (RSF congênito e adquirido) não apresentaram diferenças estatisticamente significativas em termos de idade, BUN sérico, triglicerídeos, colesterol total, lipoproteína de alta densidade, níveis de PCR, DP de peso, DP de altura, índice de massa corporal (IMC), pressão arterial, relação proteína/creatinina urinária e velocidade de hemossedimentação. No entanto, houve uma diferença estatisticamente significativa nos níveis de creatinina sérica, com níveis mais elevados observados em pacientes com RSF adquirido (p = 0,023). Isso sugere uma possível distinção na função renal entre os grupos RSF congênito e RSF adquirido (Tabela 1).

**Tabela 1:** Dados demográficos e laboratoriais de pacientes com rim solitário funcional

Parâmetros	RSF congênito (n = 41)	RSF adquirido (n = 18)	p^a,b,c^
Idade (anos)	10,12 ± 4,45	12,28 ± 4,28	0,089^a^
Sexo			
Masculino, n (%)	24 (58,5%)	7 (38,9%)	0,164^b^
Feminino, n (%)	17 (41,5%)	11 (61,1%)	
Escore-z de altura	−0,18 ± 2,48	−0,56 ± 2,09	0,57^a^
Escore-z de peso	−0,03 ± 2,05	0,16 ± 1,44	0,727^a^
Índice de massa corporal (kg/m^2^)	18,89 ± 4,85	20,95 ± 7,48	0,210^a^
Escore-z do índice de massa corporal	0,37 ± 1,39	0,21 ± 1,51	0,689^a^
PA sistólica	112,98 ± 13,14	116,11 ± 9,32	0,364^a^
PA diastólica	71,10 ± 9,75	73,39 ± 9,25	0,402^a^
Escore-z da PA sistólica	1,25 ± 1,20	1,23 ± 0,81	0,959^a^
Escore-z da PA diastólica	1,25 ± 0,87	1,13 ± 0,73	0,618^a^
Nitrogênio ureico no sangue (mg/dL)	26,63 ± 10,74	28,87 ± 21,83	0,599^a^
Creatinina sérica (mg/dL)	0,53 (0,27–1,20)	0,70 (0,49–1,62)	**0,010^c^**
TFGe (ml/min/1,73m^2^)	103,07 ± 26,00	84,78 ± 20,52	**0,011^a^**
Triglicerídeos (mg/dL)	91,78 ± 63,87	94,28 ± 52,22	0,885^a^
Colesterol total (mg/dL)	146,90 ± 28,39	161,56 ± 30,77	0,080^a^
Lipoproteína de alta densidade (mg/dl)	57,24 ± 18,42	55,94 ± 15,68	0,795^a^
Colesterol LDL (mg/dL)	75,0 (16–140)	92,50 (47–262)	**0,005^c^**
Proteína C-reativa (mg/dL)	1,74 ± 3,96	0,82 ± 0,80	0,331^a^
Velocidade de hemossedimentação (mm/h)	6,29 ± 5,49	6,11 ± 5,71	0,908^a^
Relação proteína/creatinina urinária (mg/mg)	0,16 ± 0,18	0,22 ± 0,36	0,558^a^

Abreviações – RSF: Rim solitário funcional; PA: pressão arterial; LDL: lipoproteína de baixa densidade; TFGe: taxa de filtração glomerular estimada. Notas – Os dados numéricos são apresentados como média ± DP e mediana (mínimo-máximo), enquanto os dados categóricos são apresentados como números e porcentagens. O valor de p é utilizado para comparações entre pacientes com rim solitário funcional congênito e adquirido e p < 0,05 indica significância estatística.

^a^Teste t de Student.

^b^Teste qui-quadrado ou de Pearson, quando apropriado.

^c^Teste U de Mann-Whitney.

Além disso, o CkiD-eGFR-SCr-CysC foi menor (p = 0,011) e os níveis de colesterol LDL foram mais elevados (p = 0,018) em crianças com RSF adquirido (Figura 1).

**Figura 1.** (A) Colesterol de lipoproteína de baixa densidade (LDL) (mg/dL), (B) Índice de Pulsatilidade, (C) Creatinina sérica (mg/dL), (D) taxa de filtração glomerular estimada (TFGe) (mL/min/1,73 m^2^), (E) Índices de amplificação central (%) em pacientes com rim solitário funcional congênito e rim solitário funcional adquirido.

O número de casos com hipertensão, proteinúria, declínio da TFGe e hiperfiltração, como indicadores de injúria renal, não apresentou diferença significativa entre os grupos (p > 0,005 para todos). No entanto, a hiperfiltração foi significativamente mais comum em pacientes com RSF congênito (p = 0,038) (Tabela 2).

**Tabela 2:** Parâmetros de injúria renal associados ao rim solitário funcional

Parâmetros clínicos	RSF congênito (n = 41)	RSF adquirido (n = 18)	p^a^
Hipertensão, (n, %)	19 (46,3)	4 (22,2)	0,080
Proteinúria, (n, %)	4 (9,8)	4 (22,2)	0,198
TFGe <90 mL/min/1,73m2 (n, %)	17 (41,5)	13 (72,2)	0,061
Hiperfiltração (TFGe >2DP) (n, %)	21 (51,2)	4 (22,2)	**0,038**

Abreviações – RSF: Rim solitário funcional; TFGe: taxa de filtração glomerular estimada. Notas – Os dados são apresentados em números e porcentagens. O valor de p é utilizado para as comparações entre pacientes com rim solitário funcional congênito e adquirido e p < 0,05 indica significância estatística.

^a^O teste qui-quadrado ou o teste exato de Fisher foi usado conforme apropriado.

Não houve diferença estatisticamente significativa entre os grupos em termos de valores de EIMc, VOPcf, VOP, escore-z de altura e escore-z de altura da EIMc. Verificou-se que o cAIx foi significativamente maior no grupo RSF congênito em comparação com o grupo RSF adquirido (p = 0,038) (Tabela 3).

**Tabela 3:** Parâmetros de disfunção endotelial vascular expressos em crianças com rim solitário funcional congênito e adquirido

Parâmetros doppler	RSF congênito (n = 41)	RSF adquirido (n = 18)	p^a,b^
EIMc	0,48 ± 0,08	0,51 ± 0,10	0,402^a^
VOPcf	4,55 ± 0,41	4,56 ± 0,36	0,952^a^
cAIx (%)	28,34 ± 13,88	20,67 ± 9,86	**0,038^a^**
Escore-z de altura da VOPcf	0,19 ± 0,47	0,27 ± 0,35	0,511^a^
Escore-z de altura da EIMc	2,60 ± 1,92	2,89 ± 2,29	0,624^a^
IRR	0,63 ± 0,04	0,63 ± 0,03	0,777^a^
IPR	1,19 (0,9–1,86)	1,14 (1,01–1,30)	0,236^b^

Abreviações – EIMc; Espessura da íntima-média carotídea; VOPcf: velocidade da onda de pulso carotídeo-femoral; cAIx: índice de amplificação central; IRR: índice de resistividade renal; IPR: índice de pulsatilidade da artéria renal. Notas – Os dados numéricos são apresentados como média±DP e mediana (mínimo-máximo). O valor de p é utilizado para as comparações entre pacientes com rim solitário funcional congênito e adquirido e p < 0,05 indica significância estatística.

^a^Teste t de Student.

^b^Teste U de Mann-Whitney.

Não foi encontrada correlação entre IRR e cAIx, escore-z de altura da VOPcf, escore-z de altura da EIMc, VOPcf (p = 0,236; p = 0,94; p = 0,47 e p = 0,109, respectivamente). Houve uma correlação positiva entre IPR e VOPcf (r = 0,321 e p = 0,013) (Tabela 4).

**Tabela 4:** Relação entre índice de resistividade renal, índice de pulsatilidade da artéria renal e parâmetros de disfunção endotelial vascular

Parâmetros	IRR			IPR	
	r	p^a^		r	p^a^
cAIx (%)	0,157	0,236		0,51	0,71
escore-z de altura da VOPcf	0,009	0,94		0,148	0,26
escore-z de altura da EIMc	0,96	0,47		0,071	0,59
VOPcf	0,211	0,109		0,321	0,013

Abreviações – IRR: Índice de resistividade renal; IPR: índice de pulsatilidade da artéria renal; cAIx: índice de amplificação central; VOPcf: velocidade da onda de pulso carotídeo-femoral; EIMc: espessura da íntima-média carotídea. Notas – p < 0,05 indica significância estatística.

^a^Análise de correlação de Spearman.

## Discussão

O estudo identificou rigidez arterial e indícios de dano renal associados à disfunção endotelial renal em crianças diagnosticadas com RSF congênito ou adquirido. Isso sugere uma possível ligação entre o dano microvascular e a rigidez arterial em indivíduos com RSF, destacando a importância de avaliar esses fatores em pacientes pediátricos com RSF. O estudo contribui para a compreensão das implicações vasculares e renais do RSF na população pediátrica.

No presente trabalho, constatamos que as crianças com RSF adquirido apresentaram valores mais elevados de creatinina sérica e valores mais baixos de TFGe. Os achados do estudo indicam que o declínio na TFGe foi mais significativo no grupo RSF adquirido em comparação ao grupo RSF congênito. Essa observação está alinhada com a pesquisa de Abou Jaoude et al.^17^, os quais também relataram uma relação inversa entre a TFG e o tempo de acompanhamento em pacientes com RSF adquirido, sem observar o mesmo padrão em casos de RSF congênito. As diferenças nos padrões de declínio podem sugerir mecanismos ou respostas distintas para o RSF congênito em relação aos casos adquiridos. Em nosso estudo, a TFGe foi calculada de acordo com a fórmula CkiD-eGFR-SCr-CysC, cujo uso é recomendado em pesquisas^11^. Indivíduos com RSF congênito passam por um processo de geração de néfrons adicionais em um estágio inicial, o que atenua o dano renal^18,19^. Em contrapartida, pesquisas demonstraram que, em casos de RSF adquirido, uma resposta compensatória normalmente se manifesta alguns anos após a nefrectomia^20^. Em consonância com relatos de estudos anteriores, observamos, em nosso estudo, que a TFGe foi inferior em crianças com RSF adquirido. Além disso, verificou-se que o colesterol LDL foi mais elevado nos casos com RSF adquirido. Com base nesses achados, pode-se concluir que os níveis de LDL tendem a aumentar com a idade, considerando que o grupo com RSF adquirido era, em média, mais velho.

Pacientes com RSF congênito e adquirido apresentam risco de hipertensão, hiperfiltração, azotemia e proteinúria devido à redução do número de néfrons^21^. O estudo observa que a prevalência relatada de hipertensão em pacientes com RSF é de 65%, conforme determinado pela pressão arterial normal registrada no consultório e pela monitorização ambulatorial da pressão arterial. No entanto, na presente análise, não houve diferença significativa entre os dois grupos em termos da presença de proteinúria e hipertensão. Curiosamente, a frequência de hipertensão em pacientes com RSF congênito foi maior (46%) em comparação com a literatura. Essa variação pode indicar diferenças potenciais na prevalência e características da hipertensão entre os pacientes com RSF, particularmente aqueles de origem congênita, em comparação com os achados da literatura existente. Indivíduos com RSF congênito ou adquirido são suscetíveis à hiperfiltração e ao declínio da TFGe. Esse estado de hiperfiltração se desenvolve como resultado de uma hipertrofia renal compensatória. No RSF congênito, o processo de hipertrofia renal compensatória é normalmente concluído durante o período intrauterino em cerca de 90% dos casos. No RSF adquirido, a hipertrofia compensatória ocorre após a nefrectomia^20^, embora possa não ser suficiente.

O processo de hipertrofia compensatória induz a ativação das células glomerulares, fibrose, vasoconstrição e, por fim, um declínio na TFGe. Os achados estão de acordo com o estudo *KIdney of MONofunctional Origin* (KIMONO), que também observou uma maior incidência de injúria renal no grupo RSF adquirido em comparação com o grupo RSF congênito. A injúria renal, nesse contexto, foi definida como hipertensão, proteinúria, TFGe prejudicada ou uso de medicação renoprotetora. Esse achado está de acordo com nossas observações, nas quais crianças com RSF adquirido apresentaram níveis mais elevados de creatinina sérica e um declínio mais acentuado na TFGe em comparação àquelas com RSF congênito. Esses desfechos sugerem que o processo de RSF adquirido pode contribuir para as diferenças nos desfechos e complicações renais^18^. Além disso, os pesquisadores observaram que o grupo RSF adquirido apresentava uma proporção maior de casos de TFGe prejudicada em comparação com o grupo RSF congênito^18,22^. Em conformidade com estudos anteriores, em nosso estudo, observou-se que a TFGe foi menor nos casos de RSF adquirido, o que acreditamos estar relacionado à ativação tardia dos mecanismos de adaptação do néfron. Da mesma forma, outro estudo demonstrou que as crianças com RSF apresentam morbidade particularmente elevada^23^.

O desenvolvimento de hiperfiltração em crianças com RSF congênito pode ser o resultado de rigidez arterial. Além disso, fatores como tabagismo, envelhecimento, hipercolesterolemia, hipertensão, hiperglicemia e histórico familiar de doença aterosclerótica precoce, obesidade, PCR elevada e infecção sistêmica crônica também estão associados à rigidez arterial^24^. A rigidez arterial, um fator de risco conhecido para problemas cardiovasculares, pode ser avaliada de forma não invasiva por meio de métodos como a análise da onda de pulso. O cAIx é uma medida da reflexão da onda de pulso, sendo também um preditor de eventos cardiovasculares^25^. No presente trabalho, verificou-se que o cAIx foi significativamente maior no grupo RSF congênito do que no grupo RSF adquirido.

O estudo confirma um achado relevante de outro estudo que encontrou uma associação entre o aumento da VOPcf e o declínio da função renal tanto em populações diabéticas quanto não diabéticas^25^. Isso ressalta a importância de avaliar a rigidez arterial, especialmente por meio de medidas como a VOPcf, como um indicador da saúde cardiovascular e sua possível conexão com a função renal. A rigidez arterial, medida pela VOPcf, é considerada um fator de risco para eventos cardiovasculares, e sua correlação com o declínio da função renal sugere uma relação intrincada entre a saúde cardiovascular e os desfechos renais^26^. O RSF tenta compensar o aumento do fluxo sanguíneo glomerular e a perda de função aumentando a carga de trabalho, levando à hiperfiltração, o que aumenta o risco de hipertensão, microalbuminúria e comprometimento das funções renais^27^. A VOPcf é um indicador independente de mortalidade cardiovascular e por todas as causas^28^. O estudo recomenda a VOPcf como o padrão-ouro para avaliar a rigidez arterial devido à sua aplicabilidade clínica, base fisiológica sólida e relevância para os desfechos clínicos. A VOPcf é um método confiável para avaliar a rigidez arterial, fornecendo informações valiosas sobre a saúde cardiovascular^29^.

A EIMc é um parâmetro morfológico que reflete alterações estruturais na parede arterial que podem levar à arteriosclerose. O estudo constatou que o cAIx foi significativamente maior em crianças com RSF congênito. Além disso, foi detectada uma correlação negativa significativa entre o IPR e a VOPcf. Esses achados sugerem que pode haver alterações subclínicas desfavoráveis na função arterial, particularmente em crianças com RSF congênito. O cAIx, que indica a reflexão da onda de pulso e é um preditor de eventos cardiovasculares, esteve elevado no RSF congênito, indicando uma possível rigidez arterial. A correlação positiva entre o IPR e a VOPcf sugere uma associação entre o estado microvascular renal e a rigidez arterial.

Sugere-se que o IRR e o IPR medidos por ultrassonografia Doppler reflitam a resistência vascular intrarrenal e que o aumento do IRR das artérias renais esteja associado à gravidade da aterosclerose sistêmica^30,31^. O IRR aumenta em doenças do parênquima renal. Nosso estudo incluiu pacientes sem doença do parênquima renal^32^. O IPR aumenta quando a rigidez arterial aumenta. O índice de resistividade vascular (IRR) e o índice de pulsatilidade (IPR) medem a elasticidade dos ramos vasculares arteriais. Portanto, os resultados de nosso estudo mostraram que o IPR aumentou à medida que a VOPcf (rigidez arterial) aumentou. O fato de se constatar que o índice cAIx é significativamente maior em crianças com RSF congênito pode indicar o início da rigidez arterial nessas crianças. Isso implica uma possível relação entre a rigidez arterial e a resistência microvascular nas artérias renais. À luz dessas informações, o IPR, que é um critério de avaliação mais fácil, acessível e não invasivo em pacientes pediátricos com RSF, pode se tornar um novo método de avaliação da rigidez arterial. A inclusão da VOPcf como um marcador de rigidez arterial, assim como a hipertensão, a TFG e a proteinúria, pode ser considerada no acompanhamento de pacientes pediátricos com RSF.

Embora o número de pacientes em nosso estudo tenha sido pequeno, este é o primeiro estudo na literatura que demonstra que o estado microvascular renal pode ser um indicador de rigidez arterial. No entanto, acreditamos que estudos prospectivos controlados e randomizados envolvendo grupos maiores de pacientes devam ser realizados para confirmar esses achados.

Nosso estudo apresentou algumas limitações, incluindo o pequeno número de pacientes com RSF adquirido. Uma amostra maior forneceria estimativas confiáveis da deterioração das funções renais. Além disso, a microalbuminúria, que é um indicador mais sensível de dano renal do que a proteinúria, deve ser avaliada, e a duração do RSF adquirido deve ser indicada.

Em conclusão, constatamos que alterações subclínicas desfavoráveis na função arterial em crianças com RSF têm início nos estágios iniciais da infância, mas o dano renal se desenvolve posteriormente. Além disso, o IPR está associado à disfunção endotelial vascular.
